# Mechanical and Chemical Characterization of a TiC/C System Synthesized Using a Focus Plasma Arc

**DOI:** 10.1371/journal.pone.0130836

**Published:** 2015-06-25

**Authors:** Reza Mahmoodian, M. Hamdi, M. A Hassan, Abolghasem Akbari

**Affiliations:** 1 Centre of Advanced Manufacturing and Materials Processing (AMMP), Department of Mechanical Engineering, University of Malaya, 50603, Kuala Lumpur, Malaysia; 2 Department of Research and Development, Azarin Kar Ind. Co., Industrial Zone 1, 7635168361, Kerman, Iran; 3 Faculty of Civil Engineering & Earth Resources, Universiti Malaysia Pahang, Pahang, Malaysia; 4 Department of Mechanical Engineering, Assiut University, Assiut, 71516, Egypt; Gazi University, TURKEY

## Abstract

Titanium carbide-graphite (TiC/C) composite was successfully synthesized from Ti and C starting elemental powders using self-propagating high-temperature synthesis technique in an ultra-high plasma inert medium in a single stage. The TiC was exposed to a high-temperature inert medium to allow recrystallization. The product was then characterized using field emission scanning electron microscopy (FESEM) coupled with energy dispersive X-ray analysis (EDX), X-ray diffraction (XRD), Rietveld refinement, nanoindentation, and micro-hardness to determine the product’s properties. The recorded micro-hardness of the product was 3660 HV, which is a 14% enhancement and makes is comparable to TiC materials.

## Introduction

Combustion synthesis yields in-situ composites made of primary reactive mixtures through an exothermic chemical reaction [1]. The combustion synthesis process is also known as self-propagating high-temperature synthesis (SHS). The temperature increases by the reaction to ignite and sustain a propagating combustion wave through the reactants to create the anticipated product. SHS is regarded as a high-temperature process, and an alternative, more inexpensive technique for ceramic industries to conventional ceramic processing [2]. SHS is a practical means of preparing advanced ceramics and is adaptable to synthesizing a range of technologically useful solid materials in a single processing stage compared with conventional ceramic processing [3, 4].

Titanium carbide (TiC) made from a titanium (Ti) and carbon (C) self-sustained reaction is characterized by low density, good wettability, high hardness, and chemical stability [5], and it is a typical transition metal carbide with a high melting point [6]. Titanium carbide is relatively resistant to oxidation up to 1100°C due to the formation of titanium oxides [7, 8]. These oxides also reportedly act as good solid-state lubricants [9] with excellent properties that have brought it to attention for high performance applications. Superhard materials have Vickers hardness (HV) above 40 GPA [10]. Titanium carbide-graphite composite has good friction and wear properties in oxidizing and reducing environments [11].

The hardness properties of TiC produced using single-stage SHS are not desirable because of the compact nature of the green powder. Therefore, a special plasma reaction chamber was developed to produce TiC under extreme thermal conditions and to keep the temperature high enough to allow the compacted starting powder to react, melt, and recrystallize. The TiC/C composite formed was investigated and compared with TiC/C produced in a hot inert crucible that had been made earlier by the authors [12]. The results were characterized in terms of microhardness, microstructure, and elemental compositions, while phase and mechanical properties were compared with a similar ceramic composite produced under controlled, declining inert temperatures [12]. Based on its properties, this product can be utilized as a machining insert tool, local enforcement of a secondary process such as ceramic-lined composite pipes [13, 14], and as a thin product in the super-capacitor industry [15, 16]. This finding will help understand the behavior of Ti-C systems when exposed to sudden and massive localized heat compared with gradual heat exposure and cooling in the presence of an argon gas stream.

## Materials and Methods

### Experimental Procedure

Starting powder materials of Ti (Sigma Aldrich, 300 mesh, 99.7% purity) and C (Sigma Aldrich, -1000 mesh, 99.9% purity) were dried for 8 h at 115°C. The dried powders were mixed using a planetary ball mill (Retsch PM 200) for 6 h at 20 min intervals according to eq (1). Titanium carbide reportedly has high enthalpy of formation up to -184 (ΔH (KJ/mole) [17].

$$Ti+C\to TiC\;+\;183\;kJ\;mol^{-1}$$ (1)

The milled powders were compacted into pellets at 1 GPa pressure in air at room temperature with no lubricant. The diameter of the pressure die used was 12 mm and the powders were pressed for 8 min.

A commercial TIG machine with 170 A direct current (DC) initiated an arc with two argon gas intake channels to the crucible. The TIG machine ensures current supply reliability during the experiments. The argon gas pressure was 0.15 mPa, and the active plasma prolonged discharge duration was 300 s. The electrode was then removed from the chamber and the chamber was isolated until it cooled to room temperature, while positive and low-pressure argon remained flowing into the system to reduce the chance of product oxidation. The specimen was then removed from the chamber for analysis and comparison to another specimen that was produced using the hot inert crucible method [12].

### Sample Characterization

The as-sintered samples were ground and analyzed by X-ray diffraction (XRD). XRD analysis was carried out using a rotating anode X-ray diffractometer (PANalytical Empyrean) with CuKα radiation (λ = 1.54056 Å), 45 kV operation, 40 mA, 0.026° step size and scanning rate of 0.1° s^-1^ over a 2θ range from 10° to 79°. The Rietveld refinement method was used to calculate the sample phase content [18]. Sample morphology was investigated by field emission scanning electron microscopy (FESEM) coupled with Energy Dispersive X-ray analysis (EDS) to determine the surface layer composition.

### Indentation Measurements

#### Micromechanical measurement

The surface of the compacted specimens was ground and then polished carefully in preparation for hardness measurement. The product’s mechanical properties were characterized in terms of micro-hardness. A Shimadzu microhardness tester measured the product’s hardness. Static indentation Vickers hardness (HV) of the polished samples was measured by micro-indentation technique. The indentation parameters were obtained using 4.903 N load and 10s dwell time.

#### Nanomechanical characteristics

Nanoindentation was carried out to determine the specimen’s Young’s modulus by applying the load-unload method [19–21]. The polished specimen was indented (loaded-unloaded) using a three-faced Berkovich tip by Shimadzu DUH-211S. The parameters involved were test force, loading speed, and hold time of 300 mN, 14.01 mN/sec and 5 s, respectively. The specimen was indented and the arithmetic average of three different indentations of the same level served to determine Young’s modulus. The sample’s 0.189 Poisson’s ratio was extracted from the CRC Materials Science and Engineering handbook and placed in DUH software to calculate Young’s modulus [22].

#### Fracture Toughness

The fracture toughness values were evaluated with the Antis equation [23] as follows:

$$K_{IC}=0.016{\left(\frac{E}{H}\right)}^{0.5}\left(\frac{p}{C^{1.5}}\right)$$ (2)

where *E* is the elastic modulus obtained from the nanoindentation test, *H* is the Vickers hardness (GPa), *P* is the applied load (N) and *C* is the diagonal crack length (m). The fracture toughness and hardness values were averaged for three samples with five indents per sample.

## Results and Discussion

**Fig 1:**
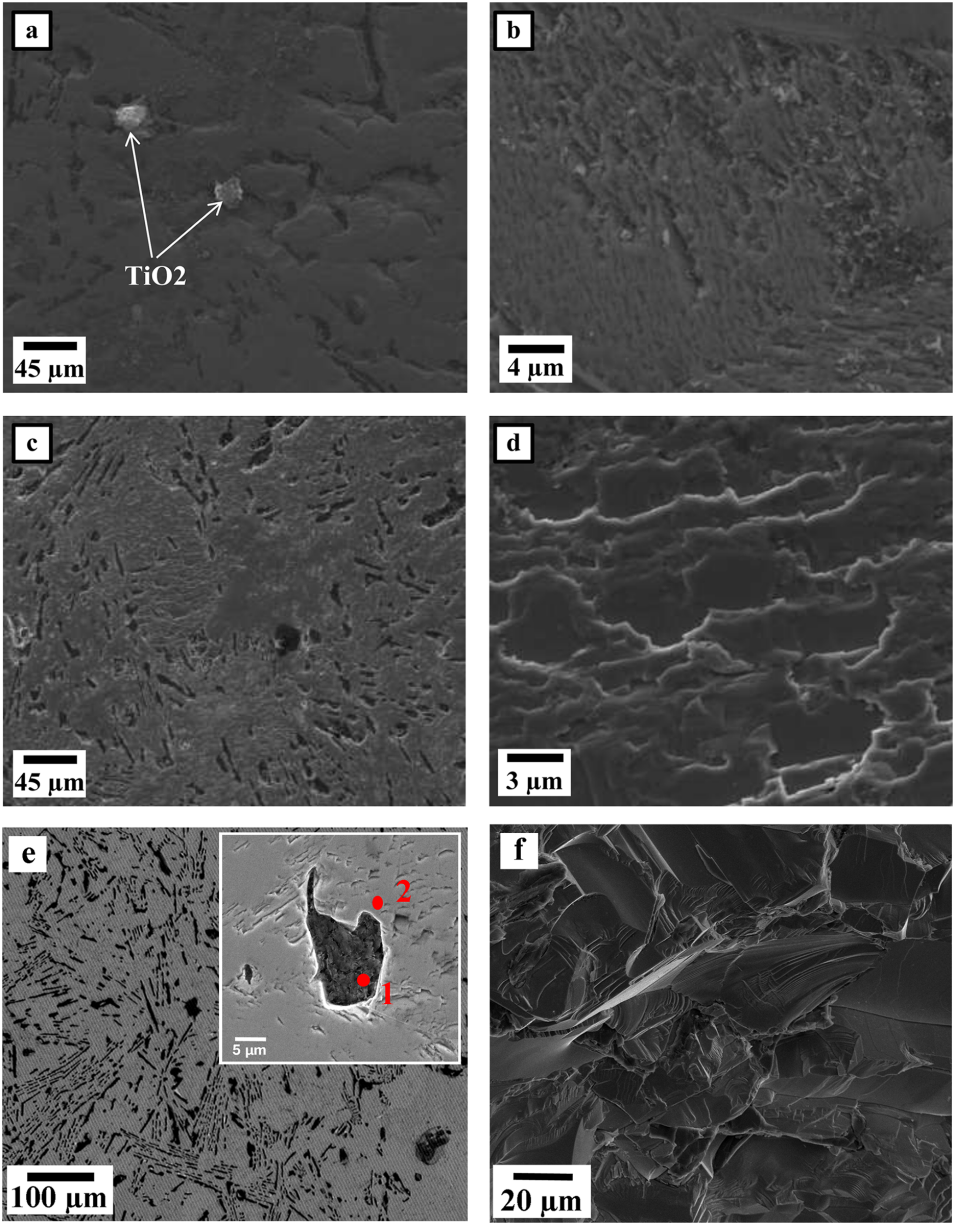
FESEM micrographs of TiC/C prepared using plasma-assisted method. (a) top surface (secondary electron image (SE)), (b) top surface at high magnification (SE) revealing the lined crystals, (c) cross section view (SE), (d) cross section view (SE) at high magnification showing the layers formed, (e) backscattered electron microscopy (BSE) image of the cross section of a typical area and its higher magnification, (f) unpolished crushed site of TiC product (SE)

**Fig 2:**
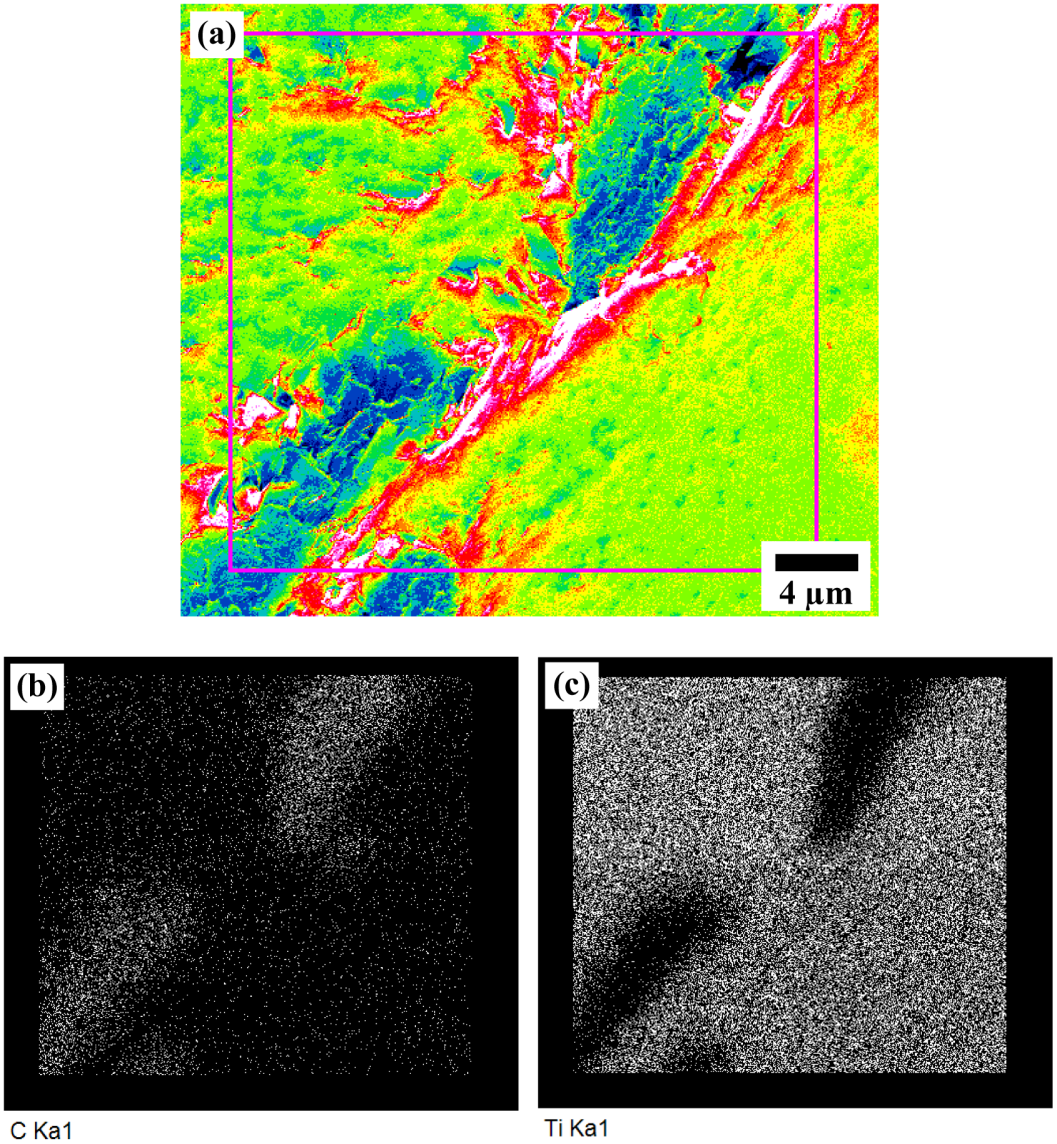
Elemental mapping spectrum of TiC/C composite cross section.

**Fig 3:**
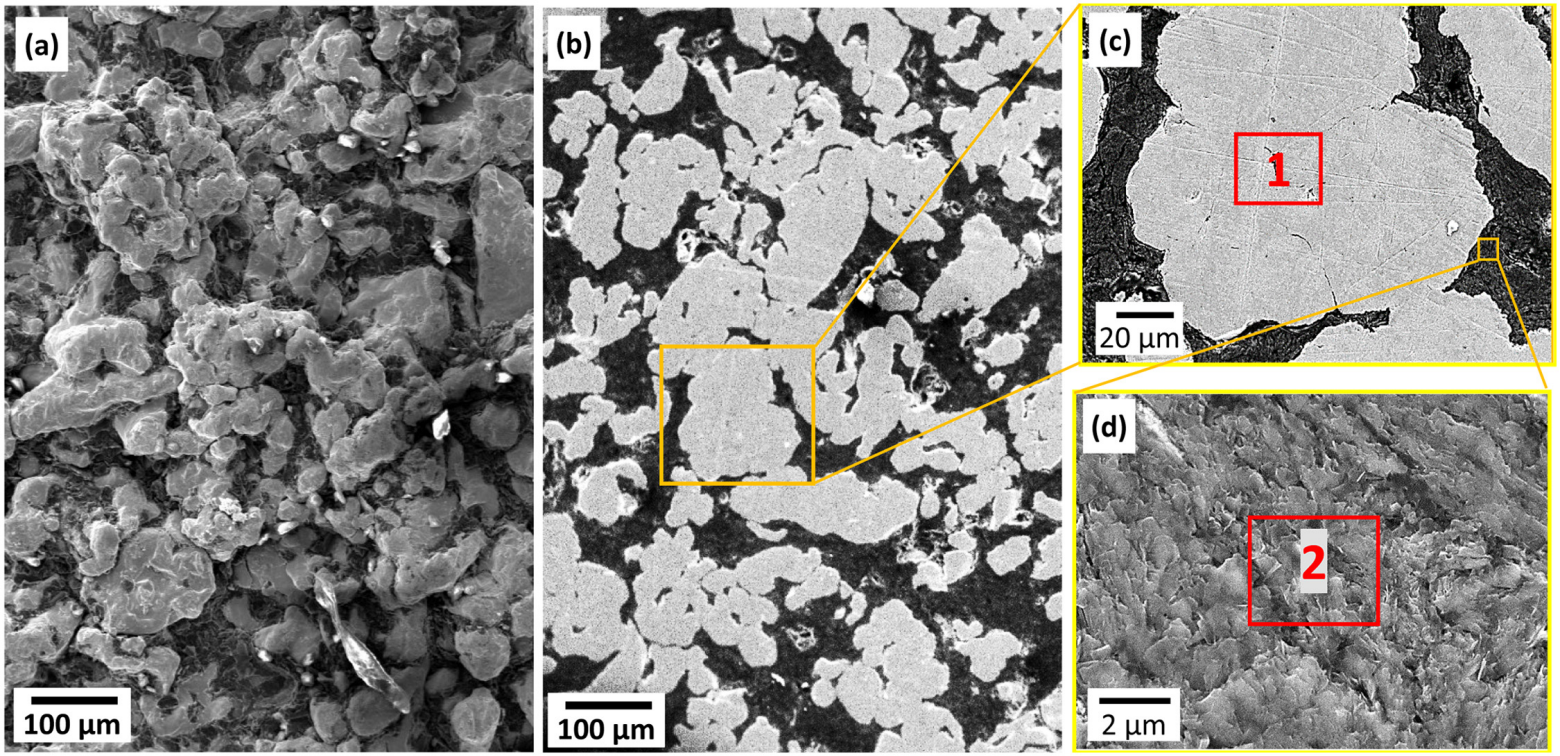
FESEM micrographs of TiC/C prepared using plasma-assisted method. (a) top surface (secondary electron image (SE)), (b) top surface at high magnification (SE) image revealing the lined crystals, (c) cross section view (SE), (d) cross section view (SE) at high magnification showing the layers formed; (e) backscattered electron microscopy (BSE) image of the cross section of a typical area and its higher magnification, (f) unpolished crushed site of TiC product (SE)

According to the macroscopic observation, the shape of the round, compacted pellet of the as-sintered product was distorted under the high-temperature plasma. No sharp edges remained, as the sample was exposed to ultra-high temperature above the melting point of TiC. The melting and boiling points of TiC are reportedly 3100°C [24]. As for the product’s microstructure, various points on the samples were studied by FESEM/EDS. Fig 1 and Fig 2 display the morphology of a typical surface area and product cross section of specimens prepared using the plasma method. The morphology of the hot-crucible prepared sample is demonstrated in Fig 3 using electron microscopy. The EDS analysis describes the relative formation of approximately uniform Ti elemental composition with C in areas of both specimens in Table 1. The corresponding sites of elemental analysis are identified in Fig 1 and Fig 3.

**Table 1:** Quantitative elemental analysis (EDS) of the samples’ cross section in plasma-assisted condition and a hot inert crucible

Element	Plasma-assisted condition (Fig 1 and 2)			Hot Crucible (Fig 3)	
	Fig 2	Point 1	Point 2	Point 1	Point 2
Ti K	49.24	0.25	45.27	77.09	-
C K	50.76	98.91	54.73	6.80	99.46
O K	-	0.84	-	16.11	0.54

Fig 1(a) represents a lower magnification of a typical point at the top surface of the polished product, (b) depicts a higher magnification of the TiC product with crystals lining the plasma zone, while (c) and (d) illustrate how the layers of molten TiC formed and solidified. Fig 1(e) is a backscattered electron microscopy micrograph of a typical area on the specimen cross section and its higher magnified micrograph image of the representing composite nature. Fig 1(f) illustrates high magnification electron microscopy of an unground, crushed specimen site.

From the micrographs in Fig 1, it is reasonable to suggest that according to eq (1) and the ultra-high temperature condition, the TiC/C product formed in the first few seconds. Thereafter, exposure to ultra-high plasma temperature consequently allowed the sample sufficient time to recrystallize and the crystals to assemble layer-by-layer, as seen in Fig 1(d).

The backscattered electron microscopy image (Fig 1(e)) of a typical area reveals how the carbon rich areas are situated in the composite. The elemental analysis given in Table 1 scales the level of each element at the site of interest. However, a carbon phase is mainly dominant in area (1), while the composition percentage in zone (2) provides nearly 45–55 (Ti-C). The higher carbon content in this zone may be due to its relative distance to the main carbon-rich zone.

Fig 2(a) represents an electron microscopy image of a typical area for distribution mapping while Fig 2(b) and 2(c) represent C and Ti elements, respectively. The elemental distribution maps of Ti and C element content were analyzed and normalized. The Ti and C elemental maps show high homogeneity and correlation between elements, indicating the presence of very small amounts of minerals. The quantitative elemental analysis (EDS) of the sample cross section corresponding to Fig 2 is given in Table 1. Evidently, the stoichiometric ratio based on the weight % of participating elements deviated from the atomic 1–1 ratio at ball-milling time after processing. This may be due to the released carbon as initiated by the electrode itself.

The FESEM micrograph of the hot inert crucible depicted in Fig 3 illustrates the poor formation of secondary compounds. Part (a) represents the secondary micrograph of an as-sintered TiC/C typical point. A backscattered electron image is illustrated in part (b), which was taken after grinding and polishing the specimen’s cross section. Part (c) shows a magnified site corresponding to part (b) with the identified EDS area given in Table 1; part (d) shows a more magnified site of the point of interest in part (c) with marked EDS analysis.

It can be understood from the micrographs of the hot inert crucible condition that TiC microstructure formed as colonies and in the middle parts, distinct unreacted carbons are clearly detected. The EDS suggests that the carbon-rich content is as much as 99% whereas the titanium rich area is around 7%.

**Fig 4:**
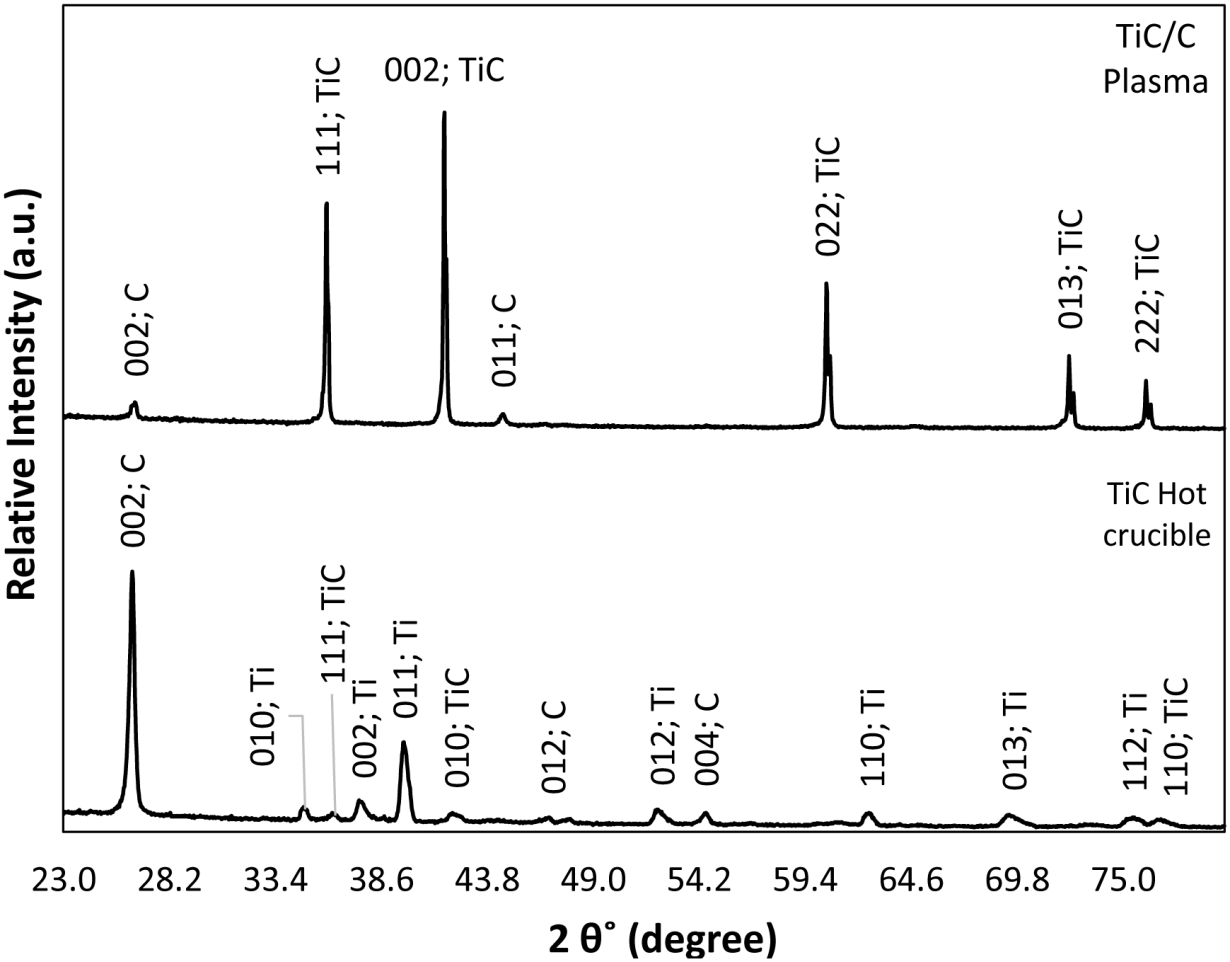
XRD diffraction patterns obtained from TiC/C plasma-assisted and hot inert crucible methods.

Fig 4 represents the X-ray diffraction pattern (XRD) of the as-sintered plasma-assisted product in comparison with the hot inert crucible sample, whose process is elaborated in recent publications by the current authors [12]. According to XRD analysis, a cubic titanium carbide structure (Khamrabaevite, JCPDS file no 96-901-2565) and hexagonal graphite structure (JCPDS file no 96-901-1578) phases were identified in the as-sintered specimen. The XRD pattern indicates sharp Bragg diffraction peaks, representing a very well crystallized product (TiC/C plasma) compared with the one prepared in non-plasma condition. The product’s phase formation evolution is apparent from the XRD analysis. However, the most intense peak of the plasma-induced product was detected at 2θ = 41.68° with a (0 0 2) plane representing TiC, while the most intense product peak in non-plasma condition was detected at 2θ = 26.38° with a (0 0 2) plane representing un-reacted carbon element.

It can be understood from the XRD pattern that the plasma-assisted method converted more of the starting powder into secondary products than the hot crucible means. The number of diffracted peaks (13 major) reduced into seven major peaks in the plasma-assisted condition. The significance of the magnitude of phase formation in this method is thus evident.

The major C peak intensity at 2θ = 26.5° of TiC in the hot crucible decreased significantly with the plasma-assisted method. Nevertheless, the Ti peaks at 2θ = 34.9, 37.6, 52.2, 62.6, 69.3, 75.5° in the hot inert crucible completely vanished in the plasma-assisted method. On the other hand, new peaks, mainly TiC appeared with the plasma-assisted method, with the most intense peaks at 2θ = 35.9, 41.7, 60.4°.

According to the XRD analysis of the plasma-assisted method, all starting Ti constituents were converted into secondary stable TiC product in the as-sintered specimen. However, very faint C peaks were observed in the XRD pattern, whose source can be attributed to the graphite electrode or crucible. According to the Rietveld method, the TiC and C content determined in this sample were 89 and 11 atomic %, respectively. The Rietveld analysis is supported by the backscattered electron microscopy micrograph in Fig 1(e), which illustrates the unreacted carbon content. Moreover, based on XRD phase analysis, the intensity of TiC phase formation with the hot inert crucible method was poorer and had broader peaks than the plasma-assisted specimen had.

The evolution of phase formation from premixed powders into secondary product in the hot inert crucible and into the final product is implicit from the XRD pattern. It provides information on the phase change and supports the notion that the reaction was not completely self-sustainable to a final product but required external heat to complete. The SEM and EDS results also support the XRD results that show how some starting material did not significantly partake in the reaction.

Micro-hardness was measured several times at various points. The hardness readings are shown in Table 2 and compared with other references. Average Vickers hardness of 3446 HV/4.903 N (33.79 GPa), maximum of 3660 HV/4.903 N (35.89 GPa) and minimum of 3250 HV/4.903 N (31.86 GPa) were recorded for the TiC/C composite sample using the plasma-assisted method. These hardness values are comparable to the superhard material classification [10]. According to XRD analysis, the hardness with the hot inert crucible method was not notable due to incomplete reaction, which is due to heat dissipation as a result of argon flow into the chamber. The argon gas cooled down the chamber and therefore, self-propagation at high temperature did not completely occur.

**Table 2:** Mechanical Properties of TiC/C compared with references

Test code	HV (GPa)	K_IC_ (MPa·m^1/2^)	Young's Modulus (GPa)	Reference
TiC/C (Plasma)	33.8–36.6	0.87	212±32	Plasma-assisted
TiC/C (Hot inert crucible)	0.56	-	65	Hot inert crucible
TiC (SPS)	25.2	4.9	410–440	[26]
TiC/TiN	16 GPa (HRA 93)	-	-	[27]
B_4_C/ TiC	32–34	3–4.5	-	[28]
TiC	31.38	1.7–3.8	439.43	[29]

Moreover, it is known that the variation in material hardness is directly correlated with the concentration of pores and phase distribution. It has also been reported that such variation can be due to the partial elastic recovery of the imprint when removing the load [25]. Therefore, the 10s dwell time was adopted in this case in order to provide sufficient relaxation time for lower elastic recovery.

By observing Fig 4 (XRD analysis), Fig 2 (elemental distribution maps) and Table 2 (mechanical properties), it can be interpreted that the unreacted C content is very well-distributed in the product while in some areas it is more concentrated, potentially due to highly localized energy transportation. The variation in specimen micro-hardness values are in agreement with SEM backscattered images and XRD. It proves that unreacted carbon content is still in the compound and formed a carbon composite. In literature, the hardness of stoichiometric titanium carbide is up to 3200 HV (31.38 GPa) [30, 31]. In the present study, the highest micro-hardness is 14% higher than reported earlier. However, fracture toughness (K_IC_ = 0.87 MPa·m^1/2^) and Young’s modulus (E_it_ = 212 GPA) have smaller values compared with the literature [26, 29]. In case of reference [28], the product is B_4_C-rich with 2–6% TiC. B_4_C is one of the hardest materials that is comparable with the current work. Therefore, the existence of crystallized C (0 0 1) and (0 1 1) planes enhances the product’s mechanical properties but not the fracture toughness.

## Conclusion

TiC/C composite with hardness similar to that of superhard materials was successfully synthesized using a developed plasma reaction chamber. The molten phases were distributed very well in the specimen, though the product was deposited layer by layer. The XRD results showed sharp, intense peaks representing highly crystallized TiC. In terms of hardness, the mechanical properties enhanced by 14% compared to other reports for TiC. The comparison between the plasma and hot inert crucible methods suggests that a self-sustaining reaction cannot occur in a positive argon atmosphere unless external heat is maintained.
